# Real-World Patterns and Outcomes of Anticoagulation Therapy in Pulmonary Embolism: An Observational Dual-Centre Registry Analysis

**DOI:** 10.3390/jcdd12100394

**Published:** 2025-10-06

**Authors:** Ivana Jurin, Josip Pejić, Karlo Gjuras, Fran Šaler, Tea-Terezija Cvetko, Nevenka Piskač Živković, Zdravko Mitrović, Šime Manola, Marin Pavlov, Aleksandar Blivajs, Kristina Marić Bešić, Dalibor Divković, Irzal Hadžibegović

**Affiliations:** 1 Department of Cardiovascular Medicine, Dubrava University Hospital, 10000 Zagreb, Croatia; ivanajurin1912@gmail.com (I.J.); fran.saler@gmail.com (F.Š.); tea.kasnik@gmail.com (T.-T.C.); sime.manola@icloud.com (Š.M.); marin.pavlov@gmail.com (M.P.); ablivajs@gmail.com (A.B.);; 2 Department of Surgery, Division of Thoracic Surgery, University Hospital Dubrava, 10000 Zagreb, Croatia; jpejic30@gmail.com; 3 Department of Family Medicine, Health Centre Bjelovar-Bilogora County, 43000 Bjelovar, Croatia; 4 Department of Internal Medicine, University Hospital Dubrava, 10000 Zagreb, Croatia; npiskac@gmail.com (N.P.Ž.); zdravmitrovic@gmail.com (Z.M.); 5 School of Medicine, University of Zagreb, 10000 Zagreb, Croatia; kmaricbesic@gmail.com; 6 Professional Undergraduate Study Physiotherapy, University North, 48000 Koprivnica, Croatia; 7 Department of Cardiovascular Diseases, University Hospital Centre Zagreb, 10000 Zagreb, Croatia; 8 Department of Surgery, University Hospital Center Osijek, 31000 Osijek, Croatia; divkovicdalibor1369@gmail.com; 9 Faculty of Medicine, University Josip Juraj Strossmayer of Osijek, 31000 Osijek, Croatia; 10 Faculty of Dental Medicine and Health Care, Josip Juraj Strossmayer University of Osijek, 31000 Osijek, Croatia

**Keywords:** pulmonary embolism, anticoagulants, direct oral anticoagulants, vitamin K antagonists, Croatia, socioeconomic factors

## Abstract

Background: Pulmonary embolism (PE) is a major cause of cardiovascular morbidity and mortality. Guidelines favor direct oral anticoagulants (DOACs) over vitamin K antagonists (VKAs), but real-world Croatian data are scarce. Methods: A prospective dual-center registry included 773 patients discharged with acute PE between 2013 and 2024. Clinical, laboratory, and socioeconomic data were collected. The primary outcome was all-cause mortality; secondary outcomes were recurrent venous thromboembolism (VTE) and major bleeding. Results: DOAC users were younger, with higher education and income, than VKA or heparin patients. Median follow-up was 1106 days. Mortality reached 60.3% with VKA, 26.0% with DOAC, and 84.1% with heparin (*p* < 0.001). VTE recurrence did not differ significantly. Major bleeding occurred in 9.3% of VKA versus 2.9% of DOAC patients (*p* = 0.003). Adjusted analysis showed a lower mortality risk with DOAC versus VKA (HR 0.62, 95% CI 0.48–0.80, *p* < 0.001), while heparin predicted higher mortality (HR 3.63, 95% CI 2.54–5.21, *p* < 0.001). Higher PESI class independently increased mortality and recurrence. Conclusion: In the first Croatian PE cohort, DOACs were linked to reduced mortality and bleeding risk compared with VKAs, with similar recurrence. Clinical, socioeconomic, and policy factors strongly influenced prescribing patterns and outcomes.

## 1. Introduction

Venous thromboembolism (VTE), encompassing deep vein thrombosis (DVT) and pulmonary embolism (PE), is the third most common acute cardiovascular condition globally, following myocardial infarction and stroke [1]. Despite advancements in treatment over the past decade, VTE remains a significant cause of morbidity and mortality, with approximately 20% of patients dying within one year of an index event [2]. Moreover, the condition is characterized by a high recurrence rate, with a 3-year cumulative incidence of up to 15% [3].

Current standard treatment for VTE includes oral anticoagulation for a duration of three to six months, guided by factors such as the presence of provoking conditions, age, and inherited or acquired risks. In cases of unprovoked VTE, extended anticoagulation beyond six months may be recommended.

Vitamin K antagonists (VKAs), such as warfarin, have long been the cornerstone of anticoagulation therapy. However, the introduction of direct oral anticoagulants (DOACs) in 2008 has transformed clinical practice. With increasing evidence supporting their efficacy and safety, DOACs have become the preferred option for many patients [4,5]. Compared to VKAs, DOACs offer advantages such as fixed dosing, rapid onset of action, and elimination of routine coagulation monitoring—benefits that may lead to cost savings and improved adherence [6].

In Croatia, four DOACs are currently available: dabigatran, rivaroxaban, apixaban, and edoxaban. Their uptake, however, varies across countries and is influenced by national guidelines and reimbursement policies. For example, DOACs rapidly surpassed VKAs in Norway by 2015, accounting for more than 80% of new anticoagulation prescriptions in VTE and atrial fibrillation patients [7]. More recent data from European registries continue to show varying adoption trends depending on policy and prescriber behavior [8].

While randomized clinical trials have demonstrated comparable efficacy and superior safety of DOACs over VKAs, real-world studies are essential to validate these outcomes across diverse patient populations, where adherence and comorbidities may differ.

In Croatia, data on real-world anticoagulant prescribing patterns for PE are limited. To date, no studies have examined the comparative use of VKAs and DOACs for PE treatment at a national level.

The objective of this study is to analyze anticoagulant prescribing patterns for acute PE in Croatia, assess compliance with contemporary guideline recommendations, and evaluate the association between treatment patterns and clinical outcomes using data from a dual-center observational registry.

## 2. Materials and Methods

### 2.1. Data Source

We maintained a prospective database of all consecutive adults (≥18 years of age) with verified acute pulmonary embolism who were admitted to two university hospital centers between December 2013 and December 2024. Data collection involved protected hospital information system entries and structured telephone interviews conducted at six-month intervals. Variables recorded included vital signs upon admission (heart rate, blood pressure, oxygen saturation, and body temperature), comorbidities (recent surgery, reduced mobility, malignancy, hypertension, diabetes mellitus, dyslipidemia, coronary artery disease, heart failure, atrial fibrillation, peripheral arterial disease, chronic obstructive pulmonary disease, chronic kidney disease, previous stroke, neurocognitive disease, and smoking status), sociodemographic factors (household income, education level, marital status, and employment status), anthropometric measurements (body mass index), laboratory parameters (blood count indices and cardiac biomarkers). Data on the type, duration, and discontinuation date of anticoagulant therapy were recorded during hospitalization and follow-up. Structured telephone questionnaires collected additional information regarding socioeconomic barriers to therapy adherence and willingness to switch therapy types under different reimbursement conditions (Supplementary Materials).

All patients provided informed consent for the use of data for research purposes. The study protocol was approved by the Ethics Committee (approval number: 2020/2409-09; date: 24 September 2020) and was conducted according to the principles outlined in the Declaration of Helsinki.

### 2.2. Pulmonary Embolism Diagnosis and Management

Pulmonary embolism diagnosis was standardized and confirmed by multi-slice computed tomography (MSCT) pulmonary angiography. Patients were treated according to the guidelines, receiving either short-acting intravenous unfractionated heparin or weight-based low-molecular-weight heparin. Post-acute and post-discharge anticoagulation therapy was managed using either VKAs (primarily warfarin), DOACs (rivaroxaban, apixaban, dabigatran, edoxaban), or heparin.

### 2.3. Study Population

**Figure 1:**
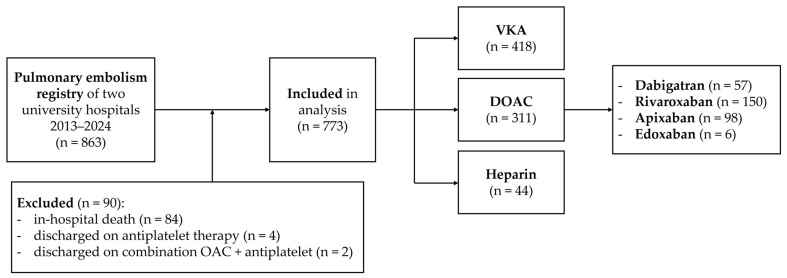
Flowchart of study process. DOAC = direct oral anticoagulant; OAC = oral anticoagulant; VKA = vitamin K antagonist.

Eligible participants included all adult patients from the registries of both centers. Patients who died during the initial hospitalization or who were discharged on antiplatelet therapy alone, or a combination of antiplatelet and anticoagulant therapy, were excluded. The remaining patients were divided into three groups based on the type of anticoagulant therapy prescribed at discharge: VKAs, DOACs, or heparin (Figure 1).

### 2.4. Primary and Secondary Outcomes

The primary outcome of the study was overall survival during follow-up, while the secondary outcomes were recurrence of VTE—either total or occurring on or after discontinuation of post-discharge anticoagulant therapy—confirmed by MSCT, and major bleeding.

### 2.5. Statistical Analysis

Categorical variables are expressed as counts (percentages). A chi-square test of independence was first conducted to assess overall differences in the distribution of categorical variables across the three study groups. If the global test indicated statistical significance (*p* < 0.05), post hoc pairwise comparisons were performed between each group combination (VKA vs. DOAC, VKA vs. heparin, and DOAC vs. heparin) using separate 2 × 2 chi-square tests. To control for type I error inflation due to multiple comparisons, a Bonferroni correction was applied to the significance threshold (α/3 = 0.0167). Therefore, pairwise differences were considered statistically significant only if the *p*-value was less than 0.0167. For significant comparisons, effect sizes were reported using Cramer’s V, interpreted as small (~0.10), medium (~0.30), or large (~0.50) associations.

Continuous variables, evaluated for normality using the Shapiro–Wilk test, demonstrated non-normal distributions and are presented as median (interquartile range; IQR). Between-group differences in continuous variables were analyzed using the Kruskal–Wallis test, with post hoc pairwise comparisons performed using the Dwass–Steel–Critchlow–Fligner method when appropriate.

Survival differences between groups were visualized with Kaplan–Meier curves and compared using the log-rank test. Independent predictors of the primary outcome (all-cause mortality during follow-up) were identified using Cox proportional hazards regression, with results expressed as hazard ratios (HRs) and 95% confidence intervals (CIs). The multivariable Cox model included type of anticoagulant therapy at discharge (VKA, DOAC, or heparin), the PESI class (I–V), and the HAS-BLED risk category (low, medium, or high/very high). Age, sex, and clinical and laboratory parameters were excluded from the model to avoid collinearity, as these are components of the PESI and HAS-BLED scores. Independent predictors of recurrent venous thromboembolism during follow-up were examined using multivariable logistic regression (odds ratios (ORs) and 95% CIs), with the same covariates entered as in the Cox model.

Statistical significance was defined as *p* < 0.05. Data were prepared in Microsoft Excel and analyzed using Jamovi (version 2.5.5; The Jamovi Project, 2024).

## 3. Results

### 3.1. Baseline Characteristics

The study included 773 patients with acute PE who were discharged on VKA (*n* = 418, 54.1%), DOAC (*n* = 311, 40.2%), or heparin (*n* = 44, 5.7%). The median age of the cohort was 72 years (IQR 60–80), with significant age differences between groups (*p* < 0.001), patients on VKA being older than those on DOAC. The proportion of male patients varied significantly (43.7% overall, *p* = 0.002), with higher male prevalence in the DOAC group compared with VKA.

Several comorbidities differed between groups. Chronic kidney disease, neurocognitive disorders, chronic obstructive pulmonary disease, and reduced mobility were significantly more prevalent among patients treated with VKAs compared with those receiving DOACs, whereas associated DVT was more common in the DOAC group (all *p* < 0.0167 after Bonferroni correction). Notably, malignancy was present in 59.1% of patients in the heparin group versus ~20% in the other groups (*p* < 0.001).

Baseline laboratory values demonstrated significant differences in hemoglobin, hematocrit, red cell distribution width, and albumin across all three treatment groups, with pairwise comparisons confirming differences between each group (all *p* < 0.05). Patients in the heparin group had higher C-reactive protein and lower albumin levels, consistent with a more severe baseline clinical profile (Table 1, Table 2 and Table 3).

**Figure 2:**
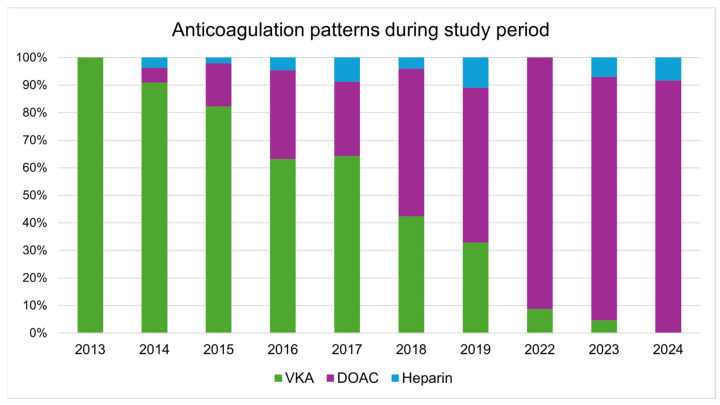
Anticoagulation patterns during study period.

**Figure 3:**
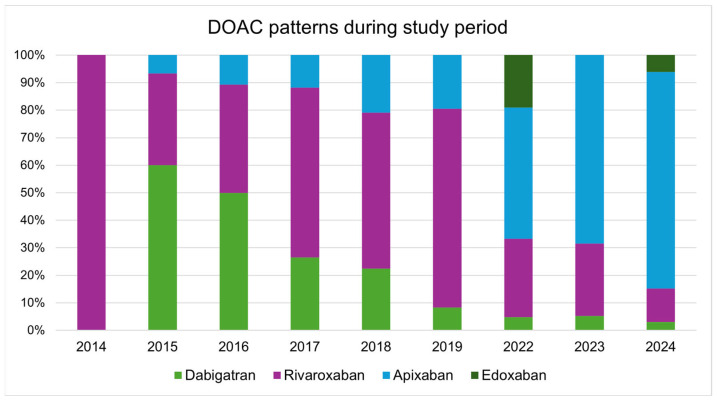
Direct oral anticoagulant (DOAC) patterns during study period.

During the study period, DOACs almost completely replaced warfarin in the treatment of pulmonary embolism (Figure 2). Whereas rivaroxaban was the most frequently prescribed DOAC in the early phase, apixaban became the predominant choice in the later years of the study period (Figure 3).

### 3.2. Sociodemographic Characteristics

Education level, marital status, employment, and household income significantly differed between groups (all *p* < 0.001). Patients discharged on DOAC were more likely to have higher education and income compared to those on VKA or heparin (Table 2 and Table 4).

### 3.3. Clinical Outcomes

**Figure 4:**
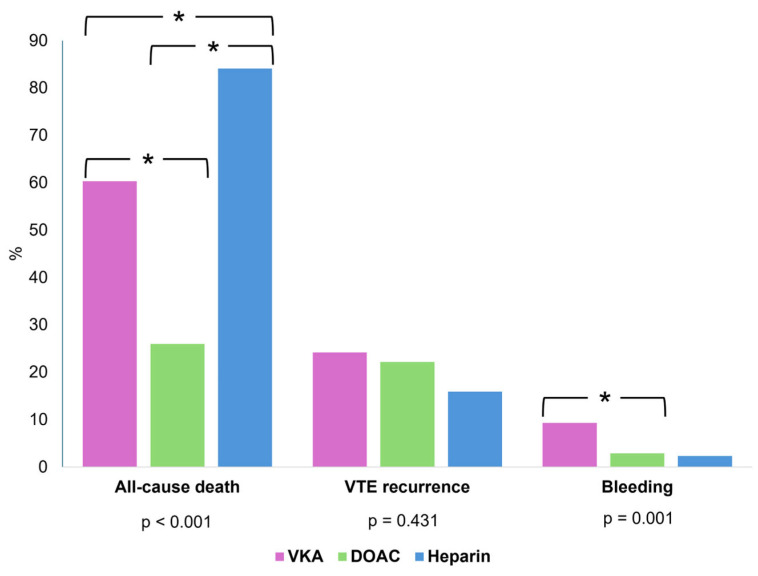
Graphical representation of clinical outcomes in pulmonary embolism patients by type of anticoagulant therapy, including post hoc analysis. Post hoc tests used Bonferroni correction with significance at * *p* < 0.0167.

The overall median follow-up duration was 1106 days. When stratified by treatment group, follow-up differed significantly (*p* < 0.001), being shortest in the heparin group (58 days), intermediate in the DOAC group (955 days), and longest in the VKA group (1390 days). All-cause mortality occurred in 47.9% of the cohort, with rates of 60.3% in the VKA group, 26.0% in the DOAC group, and 84.1% in the heparin group (*p* < 0.001). The highest malignancy-related mortality was observed in the heparin group (38.6%). The overall VTE recurrence rate was 22.9%, with no significant differences between groups (*p* = 0.431) either during anticoagulant therapy or after its discontinuation. Major bleeding events were less frequent in the DOAC group (2.9%) than in the VKA group (9.3%, *p* = 0.003; Table 5 and Figure 4). No significant differences in primary or secondary outcomes were observed between the different types of DOACs (Table 6).

### 3.4. Survival Analysis

**Figure 5:**
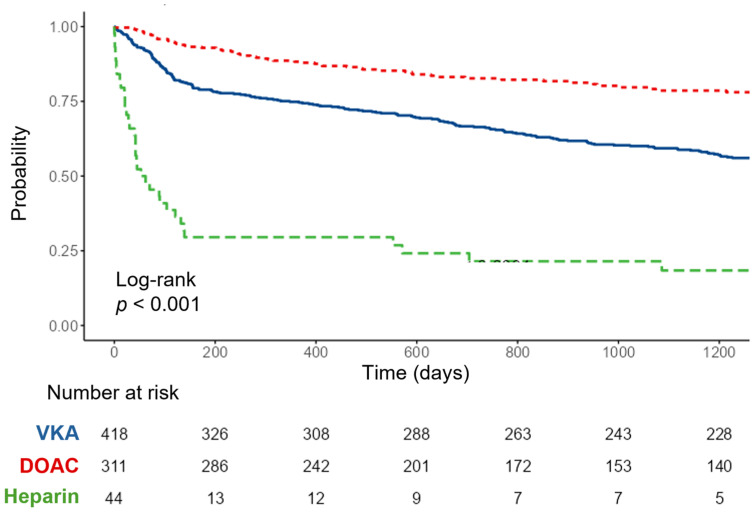
Kaplan–Meier curves showing survival probability after an index pulmonary embolism event stratified by anticoagulation type. Pairwise post hoc comparisons with Bonferroni adjustment demonstrated significant differences between all groups (all *p* < 0.001).

**Figure 6:**
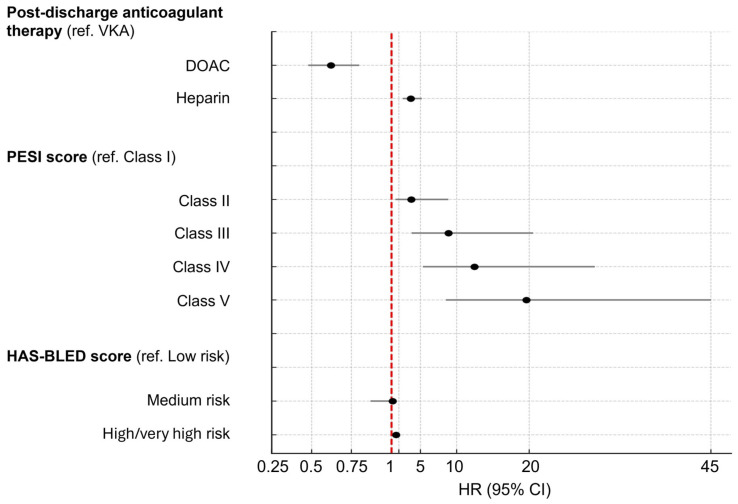
Multivariate Cox proportional hazard regression model for overall survival (forest plot).

Kaplan–Meier analysis showed significantly higher survival probability in the DOAC group compared with VKA (log-rank *p* < 0.001; Figure 5). In multivariable Cox regression, after adjusting for PESI and HAS-BLED scores, DOAC therapy was associated with a 38% lower risk of all-cause mortality compared with VKA (HR 0.62, 95% CI 0.48–0.80, *p* < 0.001). In contrast, heparin therapy was associated with a more than threefold increased mortality risk (HR 3.63, 95% CI 2.54–5.21, *p* < 0.001). Higher PESI class and high/very high HAS-BLED scores were also independently associated with worse survival (Table 7; Figure 6).

**Figure 7:**
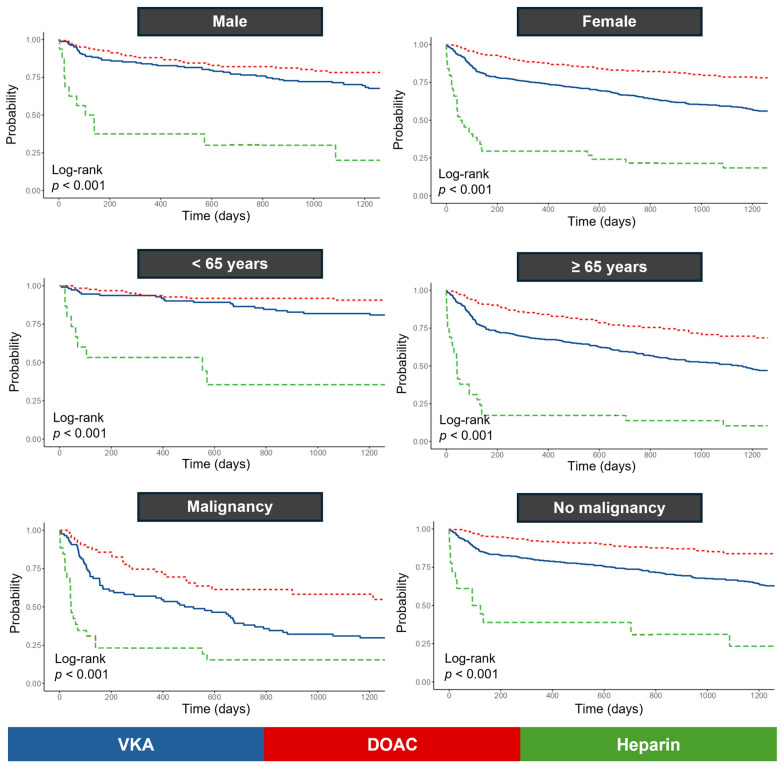
Kaplan–Meier survival curves by anticoagulant therapy (VKA, DOAC, heparin) in predefined clinical subgroups: male, female, younger (<65 years), older (≥65 years), with malignancy, and without malignancy.

Subgroup Kaplan–Meier analyses demonstrated clear separation of survival curves across all six strata (malignancy, no malignancy, male, female, ≤65 years, ≥65 years), with pairwise Bonferroni-adjusted comparisons confirming significant differences between treatment groups. In each subgroup, DOAC therapy was associated with significantly lower mortality compared with VKAs, whereas heparin consistently conferred the highest risk of death (Figure 7).

### 3.5. Predictors of VTE Recurrence

In multivariable logistic regression, PESI class V (vs. class I) was an independent predictor of recurrent VTE (OR 3.50, 95% CI 1.79–6.81, *p* < 0.001). Neither anticoagulant type nor HAS-BLED score significantly predicted recurrence (Table 8).

## 4. Discussion

Data from our real-world cohort study demonstrates substantial differences in baseline characteristics, sociodemographic profiles, and clinical outcomes among PE patients treated with VKA, DOAC, or heparin at hospital discharge. Patients discharged on DOACs tended to be younger, more frequently male, and had higher education and household income levels compared with those receiving VKAs or heparin. Conversely, the heparin group included a disproportionately high proportion of patients with active malignancy (59.1%), reduced albumin levels, elevated inflammatory markers, and markedly shorter follow-up times, suggesting advanced disease or palliative intent. The observed sociodemographic profile of DOAC users is consistent with international studies reporting that patients with higher socioeconomic status are more likely to be prescribed DOACs and to adhere to therapy [9,10]. These differences provide important insights into evolving anticoagulation prescribing patterns in Croatia and their relationship to patient outcomes.

To the best of our knowledge, no prior study representative of the Croatian healthcare system has comprehensively assessed survival and VTE recurrence patterns across different anticoagulation strategies in real-world settings. Over time, a significant increase in the use of DOACs was observed, with their use reaching 100% by the end of the enrollment period. This shift is most likely attributable to changes in reimbursement policies, as DOACs became fully covered by the Croatian Institute for Health Insurance. This mirrors trends in other European countries following reimbursement policy changes and updated guideline recommendations prioritizing DOACs for most VTE patients [4,11]. In our cohort, this change coincided with full coverage of DOAC costs by the Croatian Institute for Health Insurance, effectively removing a major economic barrier to their use.

In addition to broader availability and full reimbursement, the widespread adoption of DOACs in Croatia is also supported by growing physician confidence, driven by robust clinical evidence of their efficacy and updated international guidelines that recommend DOACs as first-line therapy for most patients with VTE [4].

Although current guidelines still recommend vitamin K antagonists (VKAs) such as warfarin in selected VTE patients, including those with severe obesity [12,13], our findings indicate that Croatian physicians increasingly prescribe DOACs even in these subpopulations, apparently without compromising clinical outcomes. Notably, earlier studies in patients with atrial fibrillation have highlighted that obesity may affect the safety and efficacy profiles of different DOACs. Specifically, dabigatran has been associated with potential loss of efficacy, while rivaroxaban may be linked to a higher risk of major bleeding in obese patients [14]. Interestingly, higher body mass index was associated with improved survival (HR 0.95, 95% CI 0.93–0.97, *p* < 0.001) in our cohort, consistent with the so-called “obesity paradox” previously described in cardiovascular diseases [15,16].

According to international guidelines, anticoagulant therapy should be continued for at least 3 to 6 months in patients with VTE provoked by transient risk factors. For those without transient risk factors and at low risk of bleeding, extended treatment beyond 6 months is recommended [4,17,18]. Extended indefinite oral anticoagulant therapy should be considered in patients with a first episode of pulmonary embolism without an identifiable provoking factor, as these patients are at increased risk of recurrence. This recommendation is consistent with the 2019 European Society of Cardiology (ESC) Guidelines, which emphasize individualized assessment of recurrence risk and bleeding risk when determining the duration of anticoagulation [4]. In our study, most patients received oral anticoagulants for longer than 6 months, reflecting a tendency among Croatian physicians to extend treatment duration.

Currently, apixaban is the most frequently prescribed DOAC in our cohort, which aligns with findings from previous research [11]. A prior study examining treatment patterns of oral anticoagulants in Croatian patients with atrial fibrillation reported no thromboembolic events in the apixaban subgroup [19]. Such findings may contribute to the perception of apixaban as a particularly “safe” option and may explain the observed preference for extended therapy in patients receiving apixaban.

Our findings from a Croatian dual-center registry align closely with results from large-scale French nationwide cohort studies [20]. The French nationwide cohort, which included over 58,000 patients with VTE between 2013 and 2018, reported substantial mortality and safety advantages with DOAC therapy, particularly apixaban, compared to VKA. In that study, apixaban use was associated with significantly lower risks of hospitalization for major bleeding, all-cause mortality, and recurrent VTE. Rivaroxaban also demonstrated a reduction in mortality, but without statistically significant reductions in bleeding or recurrence.

Taken together, both datasets consistently support the superior safety profile of DOACs over VKAs, particularly regarding the reduction in major bleeding, with at least a comparable, if not improved, effect on thromboembolic recurrence. The magnitude of mortality reduction observed in both Croatia and France suggests that the benefits of DOACs are not limited to controlled trial settings but are reproducible in real-world populations with diverse comorbidities and healthcare systems.

International guidelines have progressively evolved in favor of DOAC use. The 2016 ESC atrial fibrillation guidelines first clearly recommended DOACs over vitamin K antagonists (VKAs) for most patients with non-valvular atrial fibrillation, a position that was reaffirmed in the 2020 update and further strengthened in the 2024 ESC guidelines, as well as in the 2019 and 2023 AHA/ACC/HRS guidelines [21,22,23,24]. In Croatia, DOACs have been available since 2013, when they became widely available and partially reimbursed [19]. Nevertheless, their broader use remained limited because they were not fully reimbursed by the Croatian Health Insurance Fund until 2024. Consequently, VKAs remained the predominant oral anticoagulant in routine clinical practice. The observed delay in DOAC uptake was therefore not primarily due to lack of evidence or guideline support, but rather to reimbursement restrictions. Once this economic barrier was removed in 2024, prescribing patterns rapidly shifted in line with international guideline recommendations.

Clinical outcomes strongly favored DOAC therapy. In adjusted multivariable Cox regression, DOAC use was associated with a 38% reduction in all-cause mortality compared with VKA. Heparin therapy was associated with a more than threefold increase in mortality risk, which likely reflects selection bias, as these patients often had advanced comorbidities. Kaplan–Meier survival analysis confirmed a statistically significant survival advantage for DOAC over VKA.

The considerable differences in follow-up duration between treatment groups should be interpreted in the context of treatment selection. Shorter follow-up in the heparin group likely reflects its frequent use in patients with advanced disease or limited life expectancy, whereas longer follow-up in oral anticoagulant groups corresponds to more stable patients with better prognoses. It should also be noted that VKAs were more commonly prescribed in the earlier years of the study, which naturally resulted in longer follow-up compared with DOACs that became widely adopted later in the study period. Taken together, these factors highlight the prognostic and temporal heterogeneity of our cohort and make direct comparison of outcomes between groups more challenging.

Bleeding outcomes also supported DOAC use. Major bleeding occurred in only 2.9% of DOAC patients versus 9.3% of those on VKA, aligning with randomized trial data and meta-analyses showing lower bleeding risk with DOACs in VTE treatment [25]. VTE recurrence rates did not differ significantly between groups (*p* = 0.431), and anticoagulant type was not an independent predictor of recurrence. Instead, PESI class V emerged as the strongest predictor of recurrence risk.

In our registry, the proportion of patients experiencing recurrent VTE during ongoing anticoagulant therapy (14.5%) was notably higher than recurrence rates reported in randomized controlled trials. This finding should be interpreted in the context of our high-risk, heterogeneous “real-world” population. The most powerful independent predictor of recurrence was PESI class V, highlighting the impact of baseline disease severity on long-term outcomes. Additionally, our cohort included a substantial proportion of patients with active malignancy (22.6%), reduced mobility, chronic cardiopulmonary disease, and advanced age—each of which is independently associated with an increased risk of recurrent thromboembolism despite adequate anticoagulation. The absence of a significant association between the type of anticoagulant (VKA, DOAC, or heparin) and recurrence in multivariable analysis suggests that underlying clinical complexity and comorbidity burden outweighed the influence of pharmacological choice.

Clinical trials have also addressed the role of DOACs in patients with cancer-associated VTE. The SELECT-D trial [26] demonstrated that rivaroxaban reduced recurrent VTE compared with dalteparin, albeit with a higher incidence of clinically relevant non-major bleeding. More recently, the CARAVAGGIO trial [27] confirmed the efficacy and safety of apixaban compared with dalteparin, showing non-inferiority in preventing recurrent VTE without an excess of major gastrointestinal bleeding. These data further supported the inclusion of DOACs in the 2019 ESC guidelines as an alternative to low-molecular-weight heparin in the management of cancer-associated thrombosis [4].

In the subgroup analysis comparing different DOAC types (dabigatran, rivaroxaban, apixaban, and edoxaban), no statistically significant differences were observed in mortality, VTE recurrence, or major bleeding rates. Although numerically higher mortality was recorded in the dabigatran subgroup and higher recurrence rates in the rivaroxaban and dabigatran subgroups, these findings did not reach statistical significance. This suggests that in our population, no individual DOAC demonstrated clear superiority, which is consistent with previous reports indicating that inter-DOAC differences are generally modest and often related to patient characteristics and physician prescribing patterns rather than intrinsic drug efficacy [28]. However, the relatively small number of patients in certain subgroups, particularly edoxaban, limits interpretation and warrants cautious conclusions.

Socioeconomic and psychosocial factors can influence anticoagulant prescribing patterns. Socioeconomic analyses have shown that higher income and educational attainment are associated with a greater likelihood of receiving DOACs, whereas VKAs are more commonly prescribed for patients with lower income [9,29]. This may reflect differences in drug affordability, reimbursement coverage, and anticipated adherence, as DOACs generally require less monitoring and impose fewer dietary restrictions, which may be more manageable for patients with higher health literacy and better access to health information.

International studies support these observations. The FinACAF study demonstrated that higher income and education were independently associated with improved DOAC adherence, although persistence rates did not differ significantly by socioeconomic status [9]. In the United States, Jaladi et al. reported that high out-of-pocket costs led to DOAC discontinuation in over 8% of newly diagnosed VTE patients [29]. Socioeconomic disparities have also been noted among patients with comorbidities such as chronic kidney disease, where lower-income individuals were less likely to receive DOACs despite clinical indications [30].

Social determinants of health, particularly marital status and social support, have been linked to outcomes in VTE and other conditions. Married individuals generally exhibit better adherence, follow-up compliance, and overall survival compared to unmarried, divorced, or widowed persons [31,32]. Population-based studies from Sweden indicate that never-married, divorced, and widowed men have significantly higher all-cause and cardiovascular mortality compared with married or cohabiting men, with similar trends observed in women [32].

Our findings suggest that lower education, lower income, and lack of partner support may limit access to optimal anticoagulation therapy. These factors likely reflect differences in health literacy, ability to navigate the healthcare system, and financial barriers related to DOAC reimbursement. Until 2024, out-of-pocket costs for DOACs disproportionately affected socioeconomically disadvantaged patients, and the removal of this barrier is expected to narrow inequities. Nevertheless, disparities may persist, and targeted interventions are needed. These include simplified access pathways, patient education programs tailored to low health-literacy populations, proactive counseling and decision aids, and system-level measures such as copayment caps or monitoring of equitable prescribing. Such approaches could mitigate the impact of social determinants on clinical outcomes and promote more equitable care delivery. Taken together, these findings underscore that anticoagulant prescribing and patient outcomes are influenced by a complex interplay of socioeconomic and psychosocial factors, rather than by anticoagulant class alone.

The shift toward DOAC dominance in Croatia parallels global real-world data showing their rapid adoption due to convenience (fixed dosing, no need for routine monitoring), favorable safety profile, and comparable or superior efficacy compared to VKAs [5,33]. Of note, DOAC use in traditionally higher-risk subgroups, such as those with obesity or renal impairment, was common in our registry despite guideline caution, suggesting increasing physician confidence and reliance on individualized risk-benefit assessment.

In recent years, the introduction of Pulmonary Embolism Response Teams (PERT) has represented an important advance in the management of acute PE. These multidisciplinary teams enable rapid risk stratification and collaborative decision-making, which facilitates the timely initiation of advanced therapeutic strategies in appropriately selected patients. By expanding the availability of catheter-directed therapies and other interventional options, PERT programs have been shown to improve hemodynamic stabilization, optimize resource utilization, and potentially enhance patient outcomes [4,34].

Our study has limitations. The observational design precludes causal inference, and residual confounding is possible despite multivariable adjustment. Additionally, treatment selection in this real-world cohort was non-random and strongly shaped by clinical acuity and prognosis. Heparin was frequently chosen in patients with advanced cancer, high PESI, or anticipated short survival, which introduces confounding by indication and likely accounts for part of the mortality difference versus DOACs and VKAs. We acknowledge that we could not fully eliminate residual confounding; treatment selection (e.g., heparin in palliative or unstable patients) likely biased results toward higher mortality in that group. Differences in follow-up duration between treatment groups also limit comparability, as they reflect both prognostic heterogeneity and temporal shifts in prescribing, with VKAs used more frequently in earlier years and DOACs more commonly introduced later in the study period. Although treatment adherence is a well-established determinant of anticoagulation effectiveness, we did not have reliable data to evaluate adherence in this study, and this represents an important limitation. Finally, because our study was based on two university centers with advanced diagnostic and therapeutic capacities, the generalizability of findings to smaller or rural hospitals may be limited. Differences in case mix and availability of interventions could result in variations in treatment patterns and outcomes across the country. Although there have been initiatives and expressed intent to establish a nationwide PE/VTE registry in Croatia, this has not yet been realized due to resource and organizational constraints. The creation of such a registry would provide an invaluable platform for monitoring treatment patterns, benchmarking outcomes across diverse hospital settings, and guiding policy interventions to promote equitable access to optimal therapy.

## 5. Conclusions

In conclusion, in this large Croatian real-world cohort of PE patients, DOAC therapy was associated with improved survival, lower bleeding risk, and equivalent recurrence prevention compared with VKAs. Prescribing trends reflect the influence of updated evidence, reimbursement policies, and socioeconomic factors. Future national registry initiatives could validate these findings across a broader patient population and inform strategies to ensure equitable access to optimal anticoagulant therapy, incorporating both clinical risk stratification and social determinants of health.

## Figures and Tables

**Table 1:** Baseline characteristics of study population.

Variables	Total (*n* = 773)	VKA (*n* = 418)	DOAC (*n* = 311)	Heparin (*n* = 44)	*p*
Age (years)	72 (60–80)	74 (64–81)	69 (55–77)	72 (62–80)	<0.001
Male (%)	338 (43.7)	162 (38.8)	160 (51.4)	16 (36.4)	0.002
BMI (kg/m^2^) ^1^	28.1 (25.4–31.7)	28.3 (25.3–31.8)	27.9 (25.8–31.8)	26.0 (23.3–30.2)	0.066
Pre-admission anticoagulation (%)	186 (24.1)	113 (27.0)	66 (21.2)	7 (15.9)	0.082
Co-morbidities (%)					
Arterial hypertension	485 (62.7)	257 (61.5)	205 (65.9)	23 (52.3)	0.158
Diabetes	157 (20.3)	84 (20.1)	58 (18.6)	15 (34.1)	0.058
Coronary disease	71 (9.2%)	50 (12.0)	19 (6.1)	2 (4.5)	0.014
Heart failure	175 (22.6)	109 (26.1)	58 (18.6)	8 (18.2)	0.046
Peripheral artery disease	63 (8.2%)	41 (9.8)	15 (4.8)	7 (15.9)	0.008
Atrial fibrillation	169 (21.9)	108 (25.8)	59 (19.0)	2 (4.5)	0.001
Prior stroke	79 (10.2)	49 (11.7)	23 (7.4)	7 (15.9)	0.071
Chronic kidney disease	184 (23.8)	118 (28.2)	51 (16.4)	15 (34.1)	<0.001
Neurocognitive disease	208 (26.9)	138 (33.0)	49 (15.8)	21 (47.7)	<0.001
Chronic obstructive pulmonary disease	86 (11.1)	58 (13.9)	22 (7.1)	6 (13.6)	0.013
Malignancy	175 (22.6)	86 (20.6)	63 (20.3)	26 (59.1)	<0.001
Nicotinismus	232 (30.0)	132 (31.6)	82 (26.4)	18 (40.9)	0.084
Prior bleeding	41 (5.3)	24 (5.7)	13 (4.2)	4 (9.1)	0.333
Prior PE	46 (6.0)	24 (5.7)	19 (6.1)	3 (6.8)	0.948
Prior DVT	85 (11.0)	47 (11.2)	36 (11.6)	2 (4.5)	0.367
Surgical procedure	113 (14.6)	59 (14.1)	43 (13.8)	11 (25.0)	0.133
Reduced mobility	281 (36.4)	170 (40.7)	82 (26.4)	29 (65.9)	<0.001
Immobilized	95 (12.3)	40 (9.6)	42 (13.5)	13 (29.5)	<0.001
Provoked PE	568 (73.5)	319 (76.3)	207 (66.6)	42 (95.5)	<0.001
Associated DVT	302 (39.1)	129 (30.9)	155 (49.8)	18 (40.9)	<0.001
Clinical characteristics					
HR at admission (b.p.m.)	100 (84–115)	100 (86–115)	99 (82–111)	99 (85–112)	0.211
SBP at admission (mmHg)	125 (110–140)	123 (110–140)	130 (115–140)	120 (109–130)	0.017
Arterial oxyhemoglobin saturation (%) ^1^	93 (90–96)	93 (90–95)	94 (91–96)	94 (90–96)	0.002
Body temperature at admission (°C)	36.5 (36.3–36.9)	36.6 (36.3–36.9)	36.5 (36.2–36.9)	36.5 (36.2–36.8)	0.088
LVEF (%) ^1^	60 (51–65)	55 (45–61)	60 (55–65)	55 (51–60)	<0.001
Biological data					
White blood cell count (×10^9^/L) ^1^	9.8 (7.6–12.3)	9.8 (7.6–12.0)	9.6 (7.5–12.1)	11.8 (8.7–15.6)	0.012
Red blood cell count (×10^12^/L)	4.5 (4.1–4.9)	4.4 (4.1–4.8)	4.6 (4.2–4.9)	4.3 (3.9–4.8)	0.005
Red cell distribution width (%)	14.1 (13.3–15.3)	14.3 (13.5–15.5)	13.7 (13.0–14.9)	15.1 (14.4–16.6)	<0.001
Hemoglobin (g/L)	134 (121–146)	133 (120–144)	138 (125–148)	120 (110–136)	<0.001
Hematocrit (L/L)	0.40 (0.37–0.44)	0.40 (0.36–0.43)	0.41 (0.38–0.45)	0.38 (0.34–0.42)	<0.001
Platelet count (×10^9^/L) ^1^	226 (183–292)	231 (185–307)	219 (179–275)	237 (179–316)	0.221
Mean platelet volume (fL)	8.4 (7.7–9.2)	8.2 (7.5–9.1)	8.6 (7.9–9.4)	8.7 (8.2–10.3)	<0.001
Creatinine (µmol/L) ^1^	84 (74–102)	88 (77–107)	82 (71–94)	83 (74–104)	<0.001
C-reactive protein (mg/L)	21.4 (9.6–54.4)	22.4 (11.1–47.3)	18.4 (7.2–50.7)	54.2 (30.0–101.0)	<0.001
D-dimers (mg/L) ^1^	4.4 (3.0–8.5)	4.4 (2.7–11.0)	4.4 (3.2–4.6)	4.4 (4.1–17.4)	0.058
Fibrinogen (g/L) ^1^	3.9 (3.2–5.0)	4.0 (3.2–5.0)	3.8 (3.1–5.0)	3.8 (2.6–4.7)	0.159
Albumin (g/L) ^1^	37 (33–40)	37 (32–40)	38 (35–41)	34 (30–36)	<0.001
Estimated GFR (mL/min/1.73 m^2^)	68 (51–86)	66 (50–82)	73 (56–90)	66 (45–86)	<0.001
NT-proBNP (pg/mL) ^1^	1012 (269–4336)	378 (117–1058)	1540 (475–4756)	493 (233–8950)	0.002
Positive cardiac troponin I (%) ^1^	325 (45.0)	144 (36.8)	155 (52.9)	26 (66.7)	<0.001
Scores					
Wells’ criteria for PE	4.5 (3.0–6.5)	4.5 (3.0–6.0)	5.5 (4.0–7.0)	6.0 (4.0–7.5)	0.005
PESI	100 (77–128)	103 (79–136)	94 (73–116)	122 (105–169)	<0.001
CHA_2_DS_2_–VASc	3 (2–4)	3 (2–5)	3 (2–4)	3 (2–5)	0.028
HAS-BLED	2 (1–3)	2 (1–3)	1 (0–2)	2 (1–3)	<0.001
ATRIA	3 (1–4)	3 (1–4)	1 (0–4)	4 (3–6)	<0.001

^1^ Missing data: BMI (*n* = 1); arterial oxyhemoglobin saturation (*n* = 10); LVEF (*n* = 172); white blood cell count (*n* = 1); platelet count (*n* = 9); creatinine (*n* = 2); D-dimer (*n* = 128); fibrinogen (*n* = 268); albumin (*n* = 131); NT-proBNP (*n* = 603); positive cardiac troponin I (*n* = 50). ATRIA: Anticoagulation and Risk Factors in Atrial Fibrillation score; BMI: body mass index; CHA_2_DS_2_–VASc: congestive heart failure, hypertension, age ≥ 75 years, diabetes mellitus, prior stroke/transient ischemic attack, vascular disease, age 65–74 years, and sex category; DVT: deep vein thrombosis; HAS-BLED: hypertension, abnormal renal/liver function, stroke, bleeding history or predisposition, labile international normalized ratio, elderly, drugs/alcohol; HR: heart rate; LVEF: left ventricular ejection fraction; NT-proBNP: N-terminal pro–B-type natriuretic peptide; PE: pulmonary embolism; PESI: Pulmonary Embolism Severity Index; SBP: systolic blood pressure.

**Table 2:** Post hoc analysis of categorical variables.

Variables	VKA vs. DOAC		VKA vs. Heparin		DOAC vs. Heparin	
	*p*	Cramer’s V	*p*	Cramer’s V	*p*	Cramer’s V
Sex	<0.001	0.126	0.756	-	0.061	-
Co-morbidities						
Coronary disease	0.008	0.099	0.139	-	0.681	-
Heart failure	0.018	-	0.252	-	0.941	-
Peripheral artery disease	0.012	0.093	0.207	-	0.004	0.152
Atrial fibrillation	0.029	-	0.002	0.147	0.018	-
Chronic kidney disease	<0.001	0.139	0.414	-	0.005	0.150
Neurocognitive disease	<0.001	0.195	0.051	-	<0.001	0.265
Chronic obstructive pulmonary disease	0.004	0.108	0.965	-	0.131	-
Malignancy	0.916	-	<0.001	0.264	<0.001	0.295
Reduced mobility	<0.001	0.149	0.001	0.150	<0.001	0.281
Immobilized	0.096	-	<0.001	0.184	0.006	0.146
Provoked PE	0.004	0.108	0.003	0.136	<0.001	0.208
Associated DVT	<0.001	0.192	0.173	-	0.267	-
Biological data						
Positive cardiac troponin I	<0.001	0.160	<0.001	0.175	0.105	-
Sociodemographic data						
Monthly household income	<0.001	0.422	0.010	0.169	<0.001	0.377
Education level	<0.001	0.333	<0.001	0.248	<0.001	0.306
Marital status	<0.001	0.224	0.148	-	0.001	0.226
Employment status	<0.001	0.180	0.899	-	0.256	-
Household members	<0.001	0.271	<0.001	0.244	<0.001	0.367
Participants having underage children	0.076	-	0.006	0.164	<0.001	0.270

DVT: deep vein thrombosis; PE: pulmonary embolism. Post hoc tests used Bonferroni correction with significance at *p* < 0.0167. Significant effects include Cramer’s V values interpreted as small (~0.10), medium (~0.30), or large (~0.50).

**Table 3:** Post hoc analysis of continuous variables.

Variables	VKA vs. DOAC	VKA vs. Heparin	DOAC vs. Heparin
	*p*	*p*	*p*
Age (years)	<0.001	0.871	0.168
Clinical characteristics			
SBP at admission (mmHg)	0.076	0.389	0.042
Arterial oxyhemoglobin saturation (%)	0.001	0.649	0.774
LVEF (%)	<0.001	0.934	0.091
Biological data			
White blood cell count (×10^9^/L)	0.819	0.015	0.010
Red blood cell count (×10^12^/L)	0.010	0.566	0.107
Red cell distribution width (%)	<0.001	0.002	<0.001
Hemoglobin (g/L)	0.006	0.004	<0.001
Hematocrit (L/L)	0.039	0.019	<0.001
Mean platelet volume (fL)	<0.001	0.002	0.240
Creatinine (µmol/L)	<0.001	0.381	0.656
C-reactive protein (mg/L)	0.218	<0.001	<0.001
Albumin (g/L)	<0.001	0.009	<0.001
Estimated GFR (mL/min/1.73 m^2^)	<0.001	0.996	0.245
NT-proBNP (pg/mL)	0.001	0.608	0.816
Scores			
Wells’ criteria for PE	0.027	0.040	0.327
PESI	<0.001	<0.001	<0.001
CHA_2_DS_2_–VASc	0.024	1.000	0.417
HAS-BLED	<0.001	0.700	0.090
ATRIA	0.272	0.003	0.001

ATRIA: Anticoagulation and Risk Factors in Atrial Fibrillation score; CHA_2_DS_2_–VASc: congestive heart failure, hypertension, age ≥ 75 years, diabetes mellitus, prior stroke/transient ischemic attack, vascular disease, age 65–74 years, and sex category; HAS-BLED: hypertension, abnormal renal/liver function, stroke, bleeding history or predisposition, labile international normalized ratio, elderly, drugs/alcohol; LVEF: left ventricular ejection fraction; NT-proBNP: N-terminal pro–B-type natriuretic peptide; PE: pulmonary embolism; PESI: Pulmonary Embolism Severity Index; SBP: systolic blood pressure. Post hoc pairwise comparisons used Dwass–Steel–Critchlow–Fligner after Kruskal–Wallis.

**Table 4:** Sociodemographic characteristics of study population.

Variables	Total (*n* = 773)	VKA (*n* = 418)	DOAC (*n* = 311)	Heparin (*n* = 44)	*p*
Monthly household income (%)					<0.001
<330 €	106 (13.7)	81 (19.4)	22 (7.1)	3 (6.8)	
330–431 €	144 (18.6)	91 (21.8)	50 (16.1)	3 (6.8)	
432–832 €	118 (15.3)	53 (12.7)	58 (18.6)	7 (15.9)	
>832 €	221 (28.6)	63 (15.1)	149 (47.9)	9 (20.5)	
Did not want to share information/unknown	184 (23.8)	130 (31.1)	32 (10.3)	22 (50.0)	
Education level (%)					<0.001
No elementary school	132 (17.1)	90 (21.5)	27 (8.7)	15 (34.1)	
Elementary school graduate	217 (28.1)	155 (37.1)	57(18.3)	5 (11.4)	
High school graduate	306 (39.6)	139 (33.3)	153 (49.2)	14 (31.8)	
Bachelor’s/master’s degree	110 (14.2)	32 (7.7)	71 (22.8)	7 (15.9)	
Unknown	8 (1.0)	2 (0.5)	3 (1.0)	3 (6.8)	
Marital status (%)					<0.001
Married	440 (56.9)	211 (50.5)	210 (67.5)	19 (43.2)	
Single	64 (8.3)	27 (6.5)	33 (10.6)	4 (9.1)	
Divorced	26 (3.4)	18 (4.3)	6 (1.9)	2 (4.5)	
Widow/widower	231 (29.9)	156 (37.3)	59 (19.0)	16 (36.4)	
Unknown	12 (1.6)	6 (1.4)	3 (1.0)	3 (6.8)	
Employment status (%)					<0.001
Emplyed	163 (21.1)	63 (15.1)	92 (29.6)	8 (18.2)	
Unemployed	75 (9.7)	44 (10.5)	26 (8.4)	5 (11.4)	
Retired	514 (66.5)	297 (71.1)	188 (60.5)	29 (65.9)	
Unknown	21 (2.7)	14 (3.3)	5 (1.6)	2 (4.5)	
Household members (%)					<0.001
Living alone	139 (18.0)	84 (20.1)	44 (14.1)	11 (25.0)	
Living with spouse	272 (35.2)	128 (30.6)	136 (43.7)	8 (18.2)	
Living with spouse and children	90 (11.6)	38 (9.1)	47 (15.1)	5 (11.4)	
Retirement home	154 (19.9)	110 (26.3)	31(10.0)	13 (29.5)	
Living with children (>18 years old)	7 (0.9)	4 (1.0)	3 (1.0)	0 (0.0)	
Living with family	80 (10.3)	45 (10.8)	33 (10.6)	2 (4.5)	
Widowed with underage children	1 (0.1)	1 (0.2)	0 (0.0)	0 (0.0)	
Living with parents	19 (2.5)	4 (1.0)	15 (4.8)	0 (0.0)	
Unknown	11 (1.4)	4 (1.0)	2 (0.6)	5 (11.4)	
Participants having underage children (%)					<0.001
Has underage children	33 (4.3)	14 (3.3)	18 (5.8)	1 (2.3)	
No underage children	89 (11.5)	44 (10.5)	41 (13.2)	4 (9.1)	
Has children older than 18	630 (81.5)	348 (83.3)	249 (80.1)	33 (75.0)	
Unknown	21 (2.7)	12 (2.9)	3 (1.0)	6 (13.6)	

**Table 5:** Clinical outcomes in pulmonary embolism patients by anticoagulant therapy type.

Variables	Total (*n* = 773)	VKA (*n* = 418)	DOAC (*n* = 311)	Heparin (*n* = 44)	*p*
Outcomes					
Death from any cause (%)	370 (47.9%)	252 (60.3)	81 (26.0)	37 (84.1)	<0.001
Cause of death (%)					
Embolism	49 (6.3)	31 (7.4)	7 (2.3)	11 (25.0)	
CV/MI	32 (4.1)	22 (5.3)	9 (2.9)	1 (2.3)	
Heart failure	40 (5.2)	29 (6.9)	10 (3.2)	1 (2.3)	
Stroke	13 (1.7)	11 (2.6)	2 (0.6)	0 (0.0)	
Sepsis	82 (10.6)	59 (14.1)	19 (6.1)	4 (9.1)	
Malignancy	111 (14.4)	69 (16.5)	25 (8.0)	17 (38.6)	
Intracerebral bleeding	6 (0.8)	6 (1.4)	0 (0.0)	0 (0.0)	
Any bleeding	4 (0.5)	4 (1.0)	0 (0.0)	0 (0.0)	
External causes	6 (0.8)	5 (1.2)	0 (0.0)	1 (2.3)	
Unknown	27 (3.5)	16 (3.8)	9 (2.9)	2 (4.5)	
VTE recurrences (%)					
Total VTE recurrences	177 (22.9)	101 (24.2)	69 (22.2)	7 (15.9)	0.431
VTE recurrence on therapy	112 (14.5)	66 (15.8)	43 (13.8)	3 (6.8)	0.250
VTE recurrence after therapy	65 (8.4)	35 (8.4)	26 (8.4)	4 (9.1)	0.986
Bleeding (%)	49 (6.3)	39 (9.3)	9 (2.9)	1 (2.3)	0.001
Follow-up (days)	1106 (357–2234)	1390 (335–2583)	955 (425–1926)	58 (21–483)	<0.001

CV/MI: cardiovascular disease/myocardial infarction; VTE: venous thromboembolism. Post hoc pairwise comparisons for follow-up analysis were conducted using the Dwass–Steel–Critchlow–Fligner test following the Kruskal–Wallis test (VKA vs. DOAC: *p* = 0.014; VKA vs. Heparin: *p* < 0.001; DOAC vs. Heparin: *p* < 0.001).

**Table 6:** Clinical outcomes by DOAC type in pulmonary embolism patients.

Variables	DOAC (*n* = 311)	Dabigatran (*n* = 57)	Rivaroxaban (*n* = 150)	Apixaban (*n* = 98)	Edoxaban (*n* = 6)	*p*
Outcomes						
Death from any cause (%)	81 (26.0)	21 (36.8)	37 (24.7)	22 (22.4)	1 (16.7)	0.210
VTE recurrences						
Total VTE recurrences	69 (22.2)	14 (24.6)	37 (24.7)	17 (17.3)	1 (16.7)	0.541
VTE recurrence on therapy	43 (13.8)	5 (8.8)	27 (18.0)	10 (10.2)	1 (16.7)	0.209
VTE recurrence after therapy	26 (8.4)	9 (15.8)	10 (6.7)	7 (7.1)	0 (0.0)	0.144
Bleeding (%)	9 (2.9)	1 (1.8)	4 (2.7)	3 (3.1)	1 (16.7)	0.226

**Table 7:** Univariate and multivariate Cox proportional hazard regression model for overall survival.

Variables	Total	Univariate HR (95% CI, *p*)	Multivariate HR (95% CI, *p*)
Post-discharge anticoagulant therapy (ref. VKA)	418 (54.1)		
DOAC	311 (40.2)	0.49 (0.38–0.64, *p* < 0.001)	0.62 (0.48–0.80, *p* < 0.001)
Heparin	44 (5.7)	4.18 (2.94–5.94, *p* < 0.001)	3.63 (2.54–5.21, *p* < 0.001)
PESI score (ref. Class I) ^1^	97 (12.5)		
Class II	168 (21.7)	4.38 (1.86–10.32, *p* = 0.001)	3.75 (1.58–8.87, *p* = 0.003)
Class III	167 (21.6)	10.82 (4.72–24.82, *p* < 0.001)	8.82 (3.79–20.51, *p* < 0.001)
Class IV	130 (16.8)	18.71 (8.16–42.89, *p* < 0.001)	12.43 (5.32–29.02, *p* < 0.001)
Class V	211 (27.3)	27.36 (12.09–61.94, *p* < 0.001)	19.56 (8.50–45.00, *p* < 0.001)
HAS-BLED score (ref. Low risk) ^2^	335 (43.3)		
Medium risk	215 (27.8)	1.97 (1.50–2.58, *p* < 0.001)	1.15 (0.87–1.52, *p* = 0.331)
High/very high risk	223 (28.8)	3.27 (2.54–4.21, *p* < 0.001)	1.64 (1.26–2.13, *p* < 0.001)

^1^ PESI score: class I (0–65), class II (66–85), class III (86–105), class IV (106–125), and class V (>125). ^2^ HAS-BLED score: low risk (0–1), medium risk (2), and high/very high risk (>2). Concordance = 0.771 (SE = 0.013); R-squared = 0.346 (Max possible = 0.997). HAS-BLED: hypertension, abnormal renal/liver function, stroke, bleeding history or predisposition, labile international normalized ratio, elderly, drugs/alcohol; PESI: Pulmonary Embolism Severity Index.

**Table 8:** Univariate and multivariate logistic regression model for total VTE recurrence.

Variables	Total	Univariate OR (95% CI, *p*)	Multivariate OR (95% CI, *p*)
Post-discharge anticoagulant therapy (ref. VKA)	418 (54.1)		
DOAC	311 (40.2)	1.12 (0.79–1.58, *p* = 0.533)	1.30 (0.90–1.88, *p* = 0.155)
Heparin	44 (5.7)	1.68 (0.73–3.89, *p* = 0.223)	1.30 (0.55–3.08, *p* = 0.555)
PESI score (ref. Class I) ^1^	97 (12.5)		
Class II	168 (21.7)	0.96 (0.55–1.66, *p* = 0.877)	1.03 (0.58–1.81, *p* = 0.926)
Class III	167 (21.6)	0.95 (0.55–1.65, *p* = 0.854)	1.03 (0.57–1.85, *p* = 0.931)
Class IV	130 (16.8)	1.70 (0.92–3.16, *p* = 0.091)	1.87 (0.96–3.64, *p* = 0.067)
Class V	211 (27.3)	3.16 (1.71–5.83, *p* < 0.001)	3.50 (1.79–6.81, *p* < 0.001)
HAS-BLED score (ref. Low risk) ^2^	335 (43.3)		
Medium risk	215 (27.8)	0.89 (0.60–1.32, *p* = 0.566)	0.73 (0.48–1.13, *p* = 0.155)
High/very high risk	223 (28.8)	1.39 (0.91–2.12, *p* = 0.124)	1.08 (0.67–1.73, *p* = 0.757)

^1^ PESI score: class I (0–65), class II (66–85), class III (86–105), class IV (106–125), and class V (>125). ^2^ HAS-BLED score: low risk (0–1), medium risk (2), and high/very high risk (>2). Area under the curve = 0.644; R-squared = 0.0674. HAS-BLED: hypertension, abnormal renal/liver function, stroke, bleed-ing history or predisposition, labile international normalized ratio, elderly, drugs/alcohol; PESI: Pulmonary Embolism Severity Index.

## Data Availability

The data presented in this study is available on request from the corresponding author.

## Supplementary Materials

The following supporting information can be downloaded at: https://www.mdpi.com/article/10.3390/jcdd12100394/s1.

## Abbreviations

The following abbreviations are used in this manuscript:

CI	Confidence interval
DOAC	Direct oral anticoagulant
DVT	Deep vein thrombosis
ESC	European Society of Cardiology
HAS-BLED	Hypertension, abnormal renal/liver function, stroke, bleeding history or predisposition, labile international normalized ratio, elderly, drugs/alcohol
HR	Hazard ratio
IQR	Interquartile range
MSCT	Multi-slice computed tomography
OR	Odds ratio
PE	Pulmonary embolism
PERT	Pulmonary Embolism Response Teams
PESI	Pulmonary Embolism Severity Index
VKA	Vitamin K antagonist
VTE	Venous thromboembolism

## References

[1] Raskob G.E., Angchaisuksiri P., Blanco A.N., Büller H., Gallus A., Hunt B.J., Hylek E.M., Kakkar T.L., Konstantinides S.V., McCumber M. (2014). Thrombosis: A major contributor to global disease burden. Semin. Thromb. Hemost..

[2] Tagalakis V., Patenaude V., Kahn S.R., Suissa S. (2013). Incidence of and mortality from venous thromboembolism in a real-world population: The Q-VTE Study Cohort. Am. J. Med..

[3] Huang W., Goldberg R.J., Anderson F.A., Cohen A.T., Spencer F.A. (2016). Occurrence and predictors of recurrence after a first episode of acute venous thromboembolism: Population-based Worcester Venous Thromboembolism Study. J. Thromb. Thrombolysis.

[4] Konstantinides S.V., Meyer G., Becattini C., Bueno H., Geersing G.J., Harjola V.P., Huisman M.V., Humbert M., Jennings C.S., Jiménez D. (2020). 2019 ESC Guidelines for the diagnosis and management of acute pulmonary embolism developed in collaboration with the European Respiratory Society (ERS). Eur. Heart J..

[5] Chan N., Sobieraj-Teague M., Eikelboom J.W. (2020). Direct oral anticoagulants: Evidence and unresolved issues. Lancet.

[6] Leminen A., Pyykönen M., Tynkkynen J., Tykkyläinen M., Laatikainen T. (2019). Modeling patients’ time, travel, and monitoring costs in anticoagulation management: Societal savings achievable with the shift from warfarin to direct oral anticoagulants. BMC Health Serv. Res..

[7] Urbaniak A.M., Strøm B.O., Krontveit R., Svanqvist K.H. (2017). Prescription Patterns of Non-Vitamin K Oral Anticoagulants Across Indications and Factors Associated with Their Increased Prescribing in Atrial Fibrillation Between 2012-2015: A Study from the Norwegian Prescription Database. Drugs Aging.

[8] Aarnio E., Huupponen R., Martikainen J., Korhonen M.J. (2023). Reimbursement and use of oral anticoagulants during 2014-2022: A register-based study. Explor. Res. Clin. Soc. Pharm..

[9] Teppo K., Jaakkola J., Biancari F., Halminen O., Linna M., Haukka J., Putaala J., Tiili P., Lehtonen O., Niemi M. (2022). Association of income and educational levels with adherence to direct oral anticoagulant therapy in patients with incident atrial fibrillation: A Finnish nationwide cohort study. Pharmacol. Res. Perspect..

[10] Nathan A.S., Geng Z., Dayoub E.J., Khatana S.A.M., Eberly L.A., Kobayashi T., Pugliese S.C., Adusumalli S., Giri J., Groeneveld P.W. (2019). Racial, Ethnic, and Socioeconomic Inequities in the Prescription of Direct Oral Anticoagulants in Patients With Venous Thromboembolism in the United States. Circ. Cardiovasc. Qual. Outcomes.

[11] Ghanima W., Schultze A., Donaldson R., Brodin E., Halvorsen S., Graham S., Carroll R., Ulvestad M., Lambrelli D. (2021). Oral Anticoagulation Therapy for Venous Thromboembolism in Norway: Time Trends and Treatment Patterns. Clin. Ther..

[12] Stevens S.M., Woller S.C., Baumann Kreuziger L., Bounameaux H., Doerschug K., Geersing G.J., Huisman M.V., Kearon C., King C.S., Knighton A.J. (2021). Executive Summary: Antithrombotic Therapy for VTE Disease: Second Update of the CHEST Guideline and Expert Panel Report. Chest.

[13] Steffel J., Collins R., Antz M., Cornu P., Desteghe L., Haeusler K.G., Oldgren J., Reinecke H., Roldan-Schilling V., Rowell N. (2021). 2021 European Heart Rhythm Association Practical Guide on the Use of Non-Vitamin K Antagonist Oral Anticoagulants in Patients with Atrial Fibrillation. Europace.

[14] Li X., Zuo C., Ji Q., Xue Y., Wang Z., Lv Q. (2021). Body mass index influence on the clinical outcomes for nonvalvular atrial fibrillation patients admitted to a hospital treated with direct oral anticoagulants: A retrospective cohort study. Drug Des. Dev. Ther..

[15] Alkhalfan F., Bukhari S., Rosenzveig A., Moudgal R., Khan S.Z., Ghoweba M., Chaudhury P., Cameron S.J., Tefera L. (2024). The Obesity Mortality Paradox in Patients with Pulmonary Embolism: Insights from a Tertiary Care Center. J. Clin. Med..

[16] El-Menyar A., Asim M., Al-Thani H. (2018). Obesity Paradox in Patients With Deep Venous Thrombosis. Clin. Appl. Thromb. Hemost..

[17] Chen A., Stecker E., Warden B.A. (2020). Direct oral anticoagulant use: A practical guide to common clinical challenges. J. Am. Heart Assoc..

[18] Ageno W., Farjat A., Haas S., Weitz J.I., Goldhaber S.Z., Turpie A.G.G., Goto S., Angchaisuksiri P., Dalsgaard Nielsen J., Kayani G. (2021). Provoked versus unprovoked venous thromboembolism: Findings from GARFIELD-VTE. Res. Pract. Thromb. Haemost..

[19] Jurin I., Lucijanić M., Šakić Z., Hulak Karlak V., Atić A., Magličić A., Starčević B., Hadžibegović I. (2020). Patterns of anticoagulation therapy in atrial fibrillation: Results from a large real-life single-center registry. Croat. Med. J..

[20] Chopard R., Andarelli J.N., Humbert S., Falvo N., Morel-Aleton M., Bonnet B., Napporn G., Kalbacher E., Obert L., Degano B. (2019). Prescription patterns of direct oral anticoagulants in pulmonary embolism: A prospective multicenter French registry. Thromb. Res..

[21] Kirchhof P., Benussi S., Kotecha D., Ahlsson A., Atar D., Casadei B., Castella M., Diener H.C., Heidbuchel H., Hendriks J. (2016). 2016 ESC Guidelines for the management of atrial fibrillation developed in collaboration with EACTS. Eur. Heart J..

[22] Hindricks G., Potpara T., Dagres N., Arbelo E., Bax J.J., Blomström-Lundqvist C., Boriani G., Castella M., Dan G.A., Dilaveris P.E. (2021). 2020 ESC Guidelines for the diagnosis and management of atrial fibrillation developed in collaboration with the European Association for Cardio-Thoracic Surgery (EACTS): The Task Force for the diagnosis and management of atrial fibrillation of the European Society of Cardiology (ESC) Developed with the special contribution of the European Heart Rhythm Association (EHRA) of the ESC. Eur. Heart J..

[23] Van Gelder I.C., Rienstra M., Bunting K.V., Casado-Arroyo R., Caso V., Crijns H.J.G.M., De Potter T.J.R., Dwight J., Guasti L., Hanke T. (2024). 2024 ESC Guidelines for the management of atrial fibrillation developed in collaboration with the European Association for Cardio-Thoracic Surgery (EACTS). Eur. Heart J..

[24] January C.T., Wann L.S., Calkins H., Chen L.Y., Cigarroa J.E., Cleveland J.C., Ellinor P.T., Ezekowitz M.D., Field M.E., Furie K.L. (2019). 2019 AHA/ACC/HRS Focused Update of the 2014 AHA/ACC/HRS Guideline for the Management of Patients With Atrial Fibrillation: A Report of the American College of Cardiology/American Heart Association Task Force on Clinical Practice Guidelines and the Heart Rhythm Society. J. Am. Coll. Cardiol..

[25] Papp T., Kiss Z., Rokszin G., Fábián I., Márk L., Bagoly Z., Becker D., Merkely B., Aradi D., Dézsi C.A. (2023). Mortality on DOACs Versus on Vitamin K Antagonists in Atrial Fibrillation: Analysis of the Hungarian Health Insurance Fund Database. Clin. Ther..

[26] Young A.M., Marshall A., Thirlwall J., Chapman O., Lokare A., Hill C., Hale D., Dunn J.A., Lyman G.H., Hutchinson C. (2018). Comparison of an Oral Factor Xa Inhibitor With Low Molecular Weight Heparin in Patients with Cancer with Venous Thromboembolism: Results of a Randomized Trial (SELECT-D). J. Clin. Oncol..

[27] Agnelli G., Becattini C., Meyer G., Muñoz A., Huisman M.V., Connors J.M., Cohen A., Bauersachs R., Brenner B., Torbicki A. (2020). Apixaban for the Treatment of Venous Thromboembolism Associated with Cancer. N. Engl. J. Med..

[28] Liu Z.Y., Zhang H.X., Ma L.Y., Mu G.Y., Xie Q.F., Zhou S., Wang Z.N., Wang Z., Hu K., Xiang Q. (2022). Non-vitamin K antagonist oral anticoagulants in venous thromboembolism patients: A meta-analysis of real-world studies. BMC Cardiovasc. Disord..

[29] Joy M., Williams J., Emanuel S., Kar D., Fan X., Delanerolle G., Field B.C., Heiss C., Pollock K.G., Sandler B. (2023). Trends in direct oral anticoagulant (DOAC) prescribing in English primary care (2014–2019). Heart.

[30] Emanuel S., Field B.C., Joy M., Fan X., Williams J., Kaba R.A., Lip G.Y.H., de Lusignan S. (2025). Disparities in the care and direct-acting oral anticoagulant (DOAC) management in atrial fibrillation (AF) and chronic kidney disease (CKD) in English primary care between 2018 and 2022: Primary care sentinel network database study. Open Heart.

[31] Robles T.F., Slatcher R.B., Trombello J.M., McGinn M.M. (2014). Marital quality and health: A meta-analytic review. Psychol. Bull..

[32] Lindström M., Pirouzifard M., Rosvall M., Fridh M. (2023). Marital status and cause-specific mortality: A population-based prospective cohort study in southern Sweden. Prev. Med. Rep..

[33] Couturaud F., Schmidt J., Sanchez O., Ballerie A., Sevestre M.A., Meneveau N., Bertoletti L., Connault J., Benhamou Y., Constans J. (2025). Extended treatment of venous thromboembolism with reduced-dose versus full-dose direct oral anticoagulants in patients at high risk of recurrence: A non-inferiority, multicentre, randomised, open-label, blinded endpoint trial. Lancet.

[34] Araszkiewicz A., Jankiewicz S., Sławek-Szmyt S., Klotzka A., Grygier M., Mularek-Kubzdela T., Lesiak M. (2019). Rapid clinical and haemodynamic improvement in a patient with intermediate-high risk pulmonary embolism treated with transcatheter aspiration thrombectomy. Adv. Interv. Cardiol..

